# A novel *ADAMTSL4* compound heterozygous mutation in isolated ectopia lentis: a case report and review of the literature

**DOI:** 10.1186/s13256-023-04272-7

**Published:** 2023-12-26

**Authors:** Hengguang Wei, Xuyun Meng, Huali Qin, Xia Li

**Affiliations:** The First Affiliated Hospital of Guangxi Medical University, 6 Shuangyong Road, Qingxiu District, Nanning, Guangxi Zhuang Autonomous Region 530021 People’s Republic of China

**Keywords:** Congenital ectopia lentis, Isolated ectopia lentis, *ADAMTSL4*, Early-onset cataract, Mutation

## Abstract

**Background:**

Congenital ectopia lentis is characterized by dislocation of the lens caused by partial or complete abnormalities in the zonular fibers. It can be caused by either systemic diseases or isolated ocular diseases. Gene detection techniques can provide valuable information when an etiological diagnosis is challenging. Herein, we report the case of a six-year-old girl with a confirmed diagnosis of isolated ectopia lentis caused by a compound heterozygous *ADAMTSL4* gene mutation.

**Case presentation:**

The patient was a 6-year-old Chinese Han girl with strabismus in the right eye. Slit lamp examination revealed that the lens in the right eye was opacified and dislocated, without an ectopic pupil. Gene detection demonstrated the presence of a compound heterozygous mutation in the *ADAMTSL4* gene [c. 2270dupG (p.Gly758Trpfs *59) and *c. 2110A* > *G (p.Ser704Gly)*], and the diagnosis of isolated ectopia lentis was confirmed. She underwent lens extraction, and a sutured scleral-fixated posterior chamber intraocular lens (IOL) was placed in the right eye. The best-corrected visual acuity was 0.1 one month postoperatively.

**Conclusion:**

Gene detection plays a crucial role in diagnosing disorders with similar symptoms, such as isolated ectopia lentis and Marfan syndrome. In this study, we used whole exons sequencing to diagnose isolated ectopia lentis and identified the variant *c.2110A* > *G (p.Ser704Gly)*, which may be associated with the development of ectopia lentis and early-onset cataract in the patient. These pathogenic gene mutations have significant implications for the genetic diagnosis of congenital ectopia lentis, treatment, surveillance, and hereditary and prenatal counseling for the patient and their family members.

## Background

Congenital ectopia lentis (CEL) is a rare congenital disorder characterized by lens dislocation caused by partial or complete abnormalities of the zonular fibers. Its prevalence rate estimated in a nationwide Danish retrospective study was determined to be 6.4/100,000 [1]. It can be caused by systemic diseases such as Marfan Syndrome, Weill–Marchesani syndrome, homocystinuria, or isolated ocular diseases [2]; clinical features may overlap between these disorders. It is crucial to differentiate these disorders for the further surveillance, treatment, and counseling of these patients, especially in young children [3–5].

Isolated congenital ectopia lentis (EL) includes isolated ectopia lentis (IEL), ectopia lentis et pupillae, and aniridia. IEL, a rare inherited connective tissue disorder that can be autosomal dominant (AD) or autosomal recessive (AR), is caused by mutations in the *FBN1* (*fibrillin-1*), *LTBP2* (*latent transforming growth factor beta-1 binding protein 2*) [6], or *ADAMTSL4* (*a disintegrin and metalloproteinase with thrombospondin motifs*) gene. Mutations in the *ADAMTSL4* gene are the second most frequent cause of congenital EL after *FBN1* mutations [7–9] and have been confirmed to be a frequent cause of IEL [10, 11].

In a study conducted from January 2016 to March 2021 by Chen TH *et al.* whereby 175 Chinese probands diagnosed with congenital EL consented to the gene detection assays, 92.57% (162/175) of disease-related mutations were detected in the *FBN1* (83.43%), *CPAMD8* (1.71%), *COL4A5* (0.57%), *ADAMTSL4* (3.43%), *LTBP2* (1.71%), and *CBS* (2.29%) genes [7]. In another study performed by Guo *et al.*, the incidence of IEL in 127 patients diagnosed with CEL was 20.5% (26/127), the mutation frequency of the *ADAMTSL4* gene in CEL and IEL cases was 3.94% (5/127) and 19.2% (5/26), respectively, while the frequency of *FBN1* mutations in IEL cases was 57.7% (15/26) [12]. A common founder, *ADAMTSL4* mutation *c.2663G* > *A*, was detected in Jews of Bukharian ancestry, leading to early-onset bilateral EL and the carrier frequency was 1:48 among unrelated healthy Bukharian Jews [13]. The recurrent mutation *c.2237G* > *A; p.(Arg746His)* with a shared haplotype was a major contributor to autosomal recessive EL in the Cook Island and New Zealand Māori populations and may have a founder effect that originated from Europe [14].

Here, we report the case of a six-year-old girl with opacified, and displaced lens was confirmed IEL through physical examination, ophthalmologic examination, echocardiography and gene testing. Gene testing identified a compound heterozygous mutation in the *ADAMTSL4* gene, and this novel mutation may be implicated in the development of EL and early-onset cataract in this patient.

## Case presentation

### Patient report

**Fig. 1:**
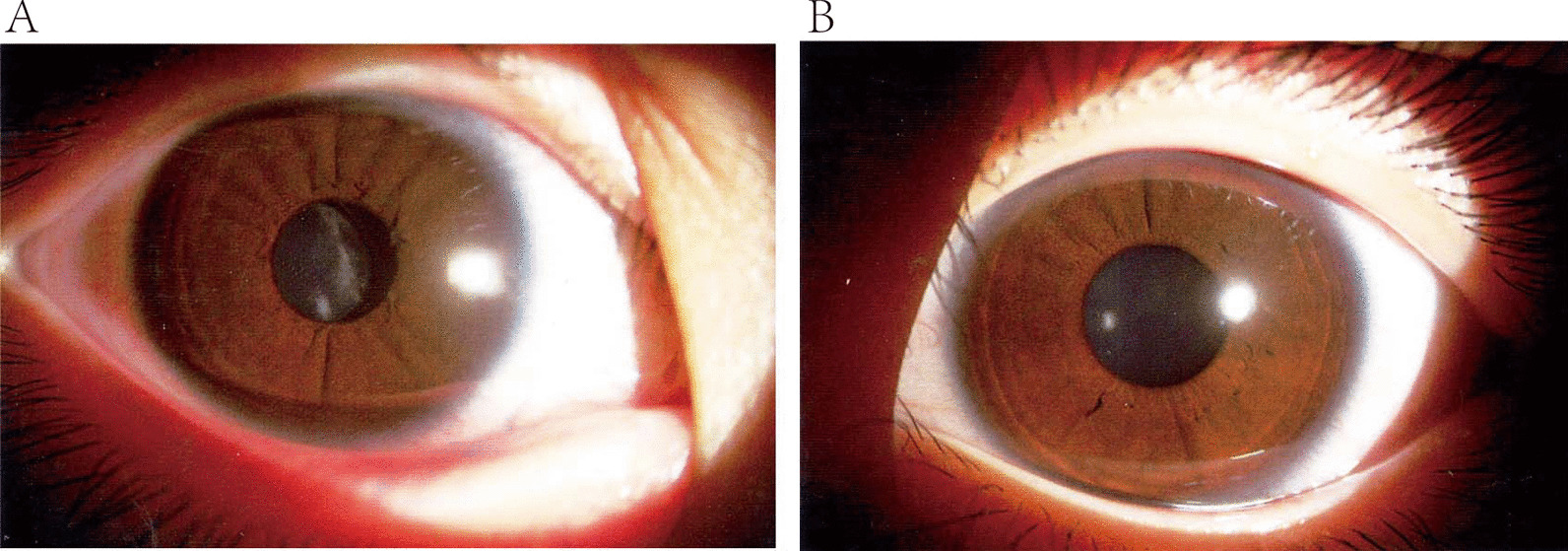
Photography of the anterior ocular segment of the patient. **A** Temporally-displaced lens with unequal white opacification in the right eye while **B** the anterior segment of the left eye was normal

The patient was a six-year-old Chinese Han girl suffering from strabismus in the right eye for three years, and thus her parents brought her to The First Affiliated Hospital of Guangxi Medical University for treatment. The child’s visual acuity was OD:HM (hand motions); OS: 0.6, and refraction was: OD: − 6.00 DS (spherical lens) − 1.75 DC (cylindrical lens) × 90 < 0.05; OS: + 0.75DC (cylindrical lens) × 100 = 1.0. The intraocular pressure measured via a non-contact tonometer was OD: 17 mmHg; OS: 18 mmHg. A slit lamp examination revealed that the sclera, conjunctiva, and cornea in the right eye were normal, but the lens was in a state of opacification and dislocation without an ectopic pupil in the anterior segment. The lens was unequal, white, opacified, and displaced to the temporal area, shown in Fig. 1. Her right eye’s posterior segment and left eye appeared normal under a slit-lamp biomicroscope and direct ophthalmoscopy.

She was tall and thin, her height was 125 cm (percentile 75–90), weight was 17.5 kg (approximately 10th percentile), and body mass index (BMI) was 11.2 kg/m^2^, indicating an extremely lean physique. A comprehensive physical examination was conducted upon admission, including temperature (36.7 °C), heart rate (80 beats/minute), respiratory rate (20 breaths/minute), and blood pressure (80/52 mmHg). Lung and heart auscultation revealed no abnormalities, and abdominal palpation did not reveal any significant findings. Neurological examination, including cranial nerve assessment, motor and sensory system examination, as well as tendon reflexes and pathological signs, all showed normal results. The arm-span measurement, wrist and thumb sign, and other skeletal features of Marfan syndrome were negative. The score of the physical examination was 0 in the systemic calculator of the revised Ghent criteria for Marfan syndrome. The patient also consented to ocular examinations, including corneal endothelium analysis, corneal topography analysis, IOL Master biometry, A-B ultrasonography, and macular optical coherence tomography. Corneal endothelium analysis displayed that the average endothelial cell density was 3285.6/mm^2^ in the right eye and 3234.8/mm^2^ in the left eye. Moreover, corneal topography analysis determined that the total corneal astigmatism was 1.4D in the right eye and 1.3D in the left eye; both were with-the-rule astigmatism. The axial length was 21.39 mm (OD) and 22.20 mm (OS) as measured by IOL Master, and 21.78 mm (OD) and 22.38 mm (OS) as detected via A ultrasonography. Neither B ultrasonography nor macular optical coherence tomography detected any abnormalities. Thereafter, she consented to an echocardiography assessment, and the results uncovered that the internal diameter of the aortic root was 20 mm, with a Z score of 2 (< 3), not satisfying the revised Ghent criteria for Marfan syndrome.

Laboratory investigations upon admission demonstrated normal values: Hematology parameters included a white blood cell count (WBC) of 9.29 × 10^9^/L, red blood cell count (RBC) of 4.74 × 10^12^/L, hemoglobin (HGB) of 133 g/L, platelet count (PLT) of 307 × 10^9^/L, neutrophil percentage (NEU%) of 0.622, and hematocrit (Hct) of 0.394. Liver function tests showed total bilirubin (TBiL) at 7.6 µmol/L, albumin (ALB) at 45 g/L, alanine aminotransferase (ALT) at 9 U/L, aspartate aminotransferase (AST) at 35 U/L, and alkaline phosphatase (ALP) at 206 U/L. Renal function parameters included urea at 3.74 mmol/L, creatinine (Cr) at 24 µmol/L, and uric acid (UA) at 196 µmol/L. Urinalysis revealed no protein, glucose, or occult blood. Electrolytes, coagulation function, and infectious disease investigations all showed normal results.

A comprehensive medical history from her parents revealed that her prenatal and developmental conditions were normal. This child’s mother had an uneventful prenatal period without specific illnesses, medication use, or exposure to harmful substances. The child was born at full term (38 weeks + 4 days) through a normal delivery, with a birth weight of 3050 g, length of 50 cm, chest circumference of 30 cm, and head circumference of 32 cm. The Apgar score was 10, and from infancy until present, there has been no history of systemic illnesses, surgeries, or prolonged medication use. The child grew up in a typical working-class family, with no history of exposure to radioactive or other harmful substances. The parents are not relatives, and none of her family members, including her elder sisters and younger brother, were diagnosed with ocular abnormalities during childhood.

The patient achieved age-appropriate developmental milestones, including rolling over at 3 months, sitting at 6 months, crawling at 9 months, walking and basic speech at 1 year and 1 month, attending kindergarten at 2 and a half years, starting first grade at 6 years with average academic performance. Nevertheless, her family observed frequent falls during her initial attempts at walking, concomitant with the identification of strabismus.

### Genetic testing

The patient and her parents were subsequently subjected to gene detection– whole-exome sequencing (WES). WES is a technique to identify pathogenic mutations by preparing DNA library, enriching and sequencing targeted genes, conducting bioinformatics analysis and selecting variants.

Genomic DNA was extracted from peripheral blood samples using MagPure Tissue&Blood DNA LQ Kit (Magen, Beijing, China) following the manufacturer’s instructions, then 200 ng genomic DNA of each individual was sheared by Biorupter (Diagenode, Belgium) to acquire 150–200 bp fragments. The ends of DNA fragment were repaired and Illumina Adaptor was added (Fast Library Prep Kit, iGeneTech, Beijing, China). After sequencing library were constructed, the whole exons were captured with AIExome Enrichment Kit V1 (iGeneTech, Beijing, China) and sequenced on Illumina platform (Illumina, San Diego, CA) with with 150 base paired‐end reads. Raw reads were filtered to remove low quality reads by using FastQC. Then clean reads were mapped to the reference genome GRCh37 by using Bwa. After removing duplications, SNV and InDel were called and annotated by using GATK. CNVkit was used to call the large copy number variations (CNVs) and the parameters were as default. To identify large copy number variations (CNVs), part of the sequencing library was sequenced directly and each sample yielded 1G Raw data. CNVs were called by using CNVseq and the control was the health parents.

## Results

**Fig. 2:**
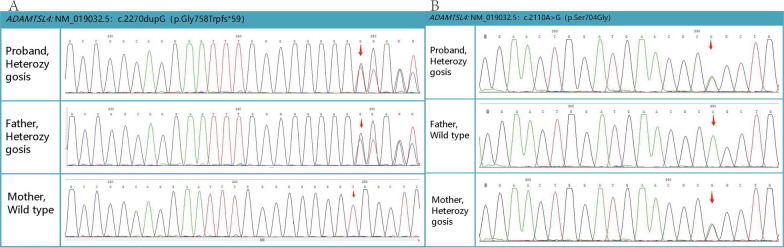
The first-generation sequencing of the patient and her parents. It confirmed that variant 1, which caused duplication in the 2270th base (G), was inherited from the father (**A**) and that variant 2, which caused a substitution in the 2110th base (A > G), was inherited from the mother (**B**)

WES identified a compound heterozygous mutation [variant 1 c. 2270dupG (P.gly758Trpfs *59) and variant 2 *c. 2110A* > *G (P.Ser704Gly)*] in the *ADAMTSL4* gene on chromosome 1 of the patient, both of which were autosomal recessive (AR) inherited mutations, shown in Table 1. The results of the first-generation sequencing validated that variant 1 was inherited from the father, whereas variant 2 was inherited from the mother, constituting a compound heterozygous mutation, shown in Fig. 2. Considering the ocular manifestations and the mutant gene, a diagnosis of IEL was made.

**Table 1:** Genetic variation results by WES

Gene	Nucleotide variation	Location	Disease	Variation classification	Genotype	variation source
ADAMTSL4	NM_019032.5 exon14 c.2270dupG p.Gly758 Trpfs*59	Chr1: 150530506	isolated ectopia lentis type 2, AR; Pupillary lens ectopic, AR	Pathogenic	Heterozygosis	Father
	NM_019032.5 exon13 c.2110A > G p.Ser704Gly	Chr1: 150530032		Unknown	Heterozygosis	Mother

*AD* autosomal dominant, *AR* autosomal recessive

In line with the ACMG criteria, *c.2270dupG* was classified as pathogenic, while *c.2110A* > *G* was designated as a variant of uncertain significance (VUS). Based on the report from WES, we learned that the variant 2 (*c.2110A* > *G*) was not detected in the Thousand Genomes Project, ExAC-EAS, gnomAD, and iGeneTechDB databases (PM2_Supporting). The variant 2 (*c.2110A* > *G*) is not recorded in the ClinVar and HGMD databases, and currently, there is no relevant literature research on *c.2110A* > *G*. Multiple computational software predict this variant to be benign to the gene or gene product (BP4). The clinical phenotype of the proband with the *c.2110A* > *G* mutation is highly consistent with Ectopia Lentis (PP4).

**Fig. 3:**
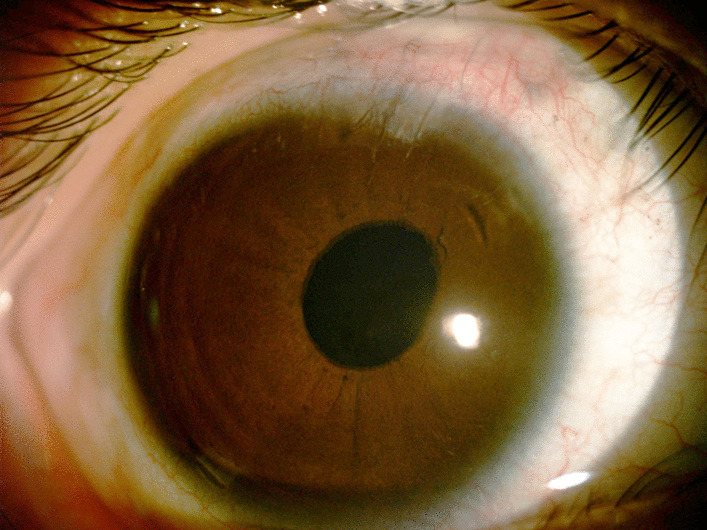
Anterior segment photograph of the patient’s right eye taken one month postoperatively

She underwent lens extraction, and a sutured scleral-fixated posterior chamber intraocular lens was placed in the right eye, and her best-corrected visual acuity was 0.1 one month postoperatively. Slit-lamp examination revealed transparent cornea, clear anterior chamber, slightly deviated pupil towards the main incision direction, and proper positioning of the intraocular lens, as shown in Fig. 3. Three months post-surgery, the uncorrected visual acuity of the child’s right eye measured 0.1, with an intraocular pressure of 14 mmHg. A slit lamp examination revealed complete healing of the conjunctival, scleral, and corneal wounds, absence of inflammation, and a stable intraocular lens without tilting or eccentricity. The refractive status of her right eye was determined to be + 0.75DS (spherical lens) − 1.25 DC (cylindrical lens) × 110, yielding a visual acuity of 0.1.

## Discussion

In this study, through WES confirmation, we diagnosed a case of IEL in a patient clinically presenting with congenital ectopia lentis combined with symptoms of early-onset cataract. Unlike Marfan syndrome, there was no necessity for the patient to undergo regular follow-ups with a cardiovascular specialist or orthopedic surgeon. Furthermore, WES revealed a novel mutation in the ADAMTSL4 gene. A comprehensive literature review and analysis led us to the conclusion that the identified variant 2 c.2110A > G (p.Ser704Gly), may potentially contribute to the development of ectopia lentis and early-onset cataracts in this particular patient.

Mutations in the *ADAMTSL4* gene can result in ocular abnormalities, including dislocation of the lens, congenital abnormalities of the iris, refractive errors that may lead to amblyopia, early-onset cataract, increased intraocular pressure, and retinal detachment [15, 16]. These mutations appear to produce the earlier manifestation of EL and are associated with increased axial length compared to *FBN1* [2]; nonetheless, an increased axial length was not noted in this case. The Guo D *et al.* study revealed that four of the five IEL probands with *ADAMTSL4* mutations also developed bilateral congenital cataracts [12]. The reported systemic findings associated with *ADAMTSL4* gene mutations include craniosynostosis, hernias, and tall stature in children, but these patients who reported systemic findings didn’t have severe skeletal or connective tissue manifestations, so *ADAMTSL4* mutations may not involve the major systemic feature of the phenotype [3].

The ADAMTS family, as modulators of microfibril formation and function in humans, comprises 19 secreted metalloproteases and 7 secreted ADAMTS-like (ADAMTSL) glycoproteins. As is well established, *ADAMTSL4* is a member of the ADAMTS family. It is located in cells and fibrillar extracellular matrix (ECM) structures of the eye, including the ciliary body, choroid, and retinal pigmented epithelium, which play a mechanical role in the eye in relation to tissue microfibrils, implying that *ADAMTSL4* participates in the formation and maintenance of the zonule [11, 17–19]. Mutations in the *ADAMTSL4* gene may cause functional abnormalities of the zonule and lead to the dislocation of the lens. Focal retinal pigment epithelium (RPE) defects were detected in homozygous Adamtsl4tvrm267 mice, primarily in the inferior eye, and the severity of the RPE phenotype was correlated with increased axial length [2, 20, 21].

When admitting a child with congenital EL, a thorough family and medical history, head-to-toe physical examination (focusing on skeletal features, dermatologic alterations, and distinct facies), ophthalmologic examination, and supplemental laboratory tests such as urine homocysteine levels and genetic testing, are required [22]. Gene detection is critical for making an etiological diagnosis, especially in indistinguishable disorders like IEL and Marfan syndrome. Panel-based Next-Generation Sequencing (NGS) strategies should be recommended as a means of detecting genetic mutations underlying complex conditions. Furthermore, a new strategy combining genetic results with clinical data is available for the early diagnosis of congenital EL, increasing the diagnostic rate from 19.43% to 40.57% [7]. The diagnostic yield of the NGS panel in a genetic cause of EL was high [in 16 out of the 24 EL patients (67%)] using the NGS gene panel, and the risk of misdiagnosis or delayed diagnosis was lowered [10].

In IEL, there are no aortic enlargement or other typical connective tissue disease features; thus, patients do not require close cardiovascular or other systemic monitoring. For this patient with an MFS-like stature, if gene detection exhibits an *FBN1* mutation, she should be classified as “potential MFS” according to the revised Ghent criteria [23, 24] and will require close cardiovascular surveillance to correct the diagnosis.

For patients with IEL and MFS, ophthalmic treatment typically involves the removal of the dislocated lens and the implantation of an artificial lens. However, in both cases, the presence of abnormal connective tissues in the patients can make ophthalmic surgeries more challenging than for regular lens dislocation cases. For this case’s patient, she will require long-term amblyopia training for the right eye after undergoing ophthalmic surgery and will need regular follow-up for potential long-term complications such as glaucoma and retinal detachment. Compared to MFS patients, the treatment for IEL patients is usually more limited and primarily focused on ophthalmic care. In contrast, MFS patients require a multidisciplinary team of doctors for management, including cardiologists, ophthalmologists, geneticists, orthopedic surgeons, particularly for the management and monitoring of the cardiovascular system. When MFS patients experience cardiac issues, they may need to undergo cardiac surgical interventions.

The WES showed a compound heterozygous mutation [variant 1 c. 2270dupG (P.gly758Trpfs *59) and variant 2 *c. 2110A* > *G (P.Ser704Gly)*] in the *ADAMTSL4* gene on chromosome 1 of the patient. Variant 1, inherited from the father, caused the *ADAMTSL4* gene to be shifted from the 758th codon and generated a premature termination codon. Variant 2, inherited from the mother, led to an amino acid substitution from serine (SER) to glycine (GLY) at the 704th position. Variants 1 and 2 constitute a compound heterozygous mutation that may lead to a partial loss of function of the *ADAMTSL4* gene, causing cell and fibrillar ECM structure abnormalities and eventually resulting in EL and early-onset cataract. Variant 1 has been reported to be pathogenic [2]. In contrast, variant 2 is novel and has not been previously reported. Mutations in heterozygous carriers were not observed to cause IEL, signifying that the pathogenesis of IEL was correlated with the effect of the gene dosage and patients with two defective alleles of the *ADAMTSL4* gene may be more susceptible to disease [2]. In the study conducted by Guo *et al.*, 12.5% (1/8) novel mutations were homozygous mutations, while 87.5% (7/8) were compound heterozygous mutations in five Chinese families; these novel mutations seem to be deleterious in humans, and heterozygous *ADAMTSL4* mutations may be commonly encountered in IEL [12]. The mutation types comprised three missense (37.5%), three frameshift (37.5%), one stopgain (12.5%), and one spicing mutations (12.5%) [12]. Although variant 2 does not result in frameshift and truncated proteins, considering the AR inheritance pattern and the findings of previous studies, we assume that variant 2 *c. 2110A* > *G (p.Ser704Gly)* may be implicated in the development of EL and early-onset cataract in this patient.

## Conclusion

Gene detection plays a crucial role in establishing the underlying cause of certain disorders, especially when the symptoms are similar, such as in the case of isolated ectopia lentis and Marfan syndrome. In this study, we employed whole exons sequencing to diagnose isolated ectopia lentis and successfully identified a variant, *c.2110A* > *G (p.Ser704Gly)*, which may be linked to the development of ectopia lentis and early-onset cataract in the patient. The discovery of these specific pathogenic gene mutations not only enhances the genetic diagnosis of CEL but also provides valuable insights for the patient’s ongoing treatment, surveillance, as well as hereditary and prenatal counseling for both the patient and their family members.

## Data Availability

The data that support the findings of this study are openly available in NCBI at https://www.ncbi.nlm.nih.gov/, accession number: PRJNA883935.

## References

[1] Fuchs J, Rosenberg T (1998). Congenital ectopia lentis. A Danish national survey. Acta Ophthalmol Scand.

[2] Zhou XM, Wang Y, Zhao L, Yu WH, Fan N, Yan NH (2015). Novel compound heterozygous mutations identified in ADAMTSL4 gene in a Chinese family with isolated ectopia lentis. Acta Ophthalmol.

[3] Neuhann TM, Stegerer A, Riess A, Blair E, Martin T, Wieser S (2015). ADAMTSL4-associated isolated ectopia lentis: further patients, novel mutations and a detailed phenotype description. Am J Med Genet Part A.

[4] Aragon-Martin JA, Ahnood D, Charteris DG, Saggar A, Nischal KK, Comeglio P (2010). Role of ADAMTSL4 mutations in FBN1 mutation-negative ectopia lentis patients. Hum Mutat.

[5] Neuhann TM (2015). Hereditary ectopia lentis. Klin Monbl Augenheilkd.

[6] Kumar A, Duvvari MR, Prabhakaran VC, Shetty JS, Murthy GJ, Blanton SH (2010). A homozygous mutation in LTBP2 causes isolated microspherophakia. Hum Genet.

[7] Chen TH, Chen ZX, Zhang M, Chen JH, Deng M, Zheng JL (2021). Combination of panel-based next-generation sequencing and clinical findings in congenital ectopia lentis diagnosed in Chinese patients. Am J Ophthalmol.

[8] Zhang L, Lai Y, Capasso J, Han S, Levin A (2015). Early onset ectopia lentis due to a FBN1 mutation with non-penetrance. Am J Med Genet A.

[9] Li J, Jia X, Li S, Fang S, Guo X (2014). Mutation survey of candidate genes in 40 Chinese patients with congenital ectopia lentis. Mol Vis.

[10] Overwater E, Floor K, van Beek D, de Boer K, van Dijk T, Hilhorst-Hofstee Y (2017). NGS panel analysis in 24 ectopia lentis patients; a clinically relevant test with a high diagnostic yield. Eur J Med Genet.

[11] Ahram D, Sato TS, Kohilan A, Tayeh M, Chen S, Leal S (2009). A homozygous mutation in ADAMTSL4 causes autosomal-recessive isolated ectopia lentis. Am J Hum Genet.

[12] Guo D, Yang F, Zhou Y, Zhang X, Cao Q, Jin G (2022). Novel ADAMTSL4 gene mutations in Chinese patients with isolated ectopia lentis. Br J Ophthalmol.

[13] Reinstein E, Smirin-Yosef P, Lagovsky I, Davidov B, Peretz Amit G, Neumann D (2016). A founder mutation in ADAMTSL4 causes early-onset bilateral ectopia lentis among Jews of Bukharian origin. Mol Genet Metab.

[14] van Bysterveldt KA, Al Taie R, Ikink W, Oliver VF, Vincent AL (2017). ADAMTSL4 assessment in ectopia lentis reveals a recurrent founder mutation in Polynesians. Ophthalmic Genet.

[15] Rødahl E, Mellgren AEC, Boonstra NE, Knappskog PM. ADAMTSL4-Related Eye Disorders. In: Adam MP, Ardinger HH, Pagon RA, Wallace SE, Bean LJH, Gripp KW, et al., editors. GeneReviews (®). Seattle: University of Washington, Seattle. 22338190

[16] Copyright © 1993–2022, University of Washington, Seattle. GeneReviews is a registered trademark of the University of Washington, Seattle. All rights reserved. 1993.

[17] Chandra A, Aragon-Martin JA, Hughes K, Gati S, Reddy MA, Deshpande C (2012). A genotype-phenotype comparison of ADAMTSL4 and FBN1 in isolated ectopia lentis. Invest Ophthalmol Vis Sci.

[18] Gabriel LA, Wang LW, Bader H, Ho JC, Majors AK, Hollyfield JG (2012). ADAMTSL4, a secreted glycoprotein widely distributed in the eye, binds fibrillin-1 microfibrils and accelerates microfibril biogenesis. Invest Ophthalmol Vis Sci.

[19] Chandra A, Jones M, Cottrill P, Eastlake K, Limb GA, Charteris DG (2013). Gene expression and protein distribution of ADAMTSL-4 in human iris, choroid and retina. Br J Ophthalmol.

[20] Hubmacher D, Apte SS (2015). ADAMTS proteins as modulators of microfibril formation and function. Matrix Biol.

[21] Collin GB, Hubmacher D, Charette JR, Hicks WL, Stone L, Yu M (2015). Disruption of murine Adamtsl4 results in zonular fiber detachment from the lens and in retinal pigment epithelium dedifferentiation. Hum Mol Genet.

[22] Mead TJ, Apte SS (2018). ADAMTS proteins in human disorders. Matrix Biol.

[23] Safi M, Nejad SK, O’Hara M, Shankar SP (2019). Ectopia lentis et pupillae caused by ADAMTSL4 pathogenic variants and an algorithm for work-up. J Pediatr Ophthalmol Strabismus.

[24] Loeys BL, Dietz HC, Braverman AC, Callewaert BL, De Backer J, Devereux RB (2010). The revised Ghent nosology for the Marfan syndrome. J Med Genet.

[25] Chandra A, Patel D, Aragon-Martin JA, Pinard A, Collod-Béroud G, Comeglio P (2015). The revised ghent nosology; reclassifying isolated ectopia lentis. Clin Genet.

